# Effect of β-anhydroicaritin on the expression levels of tumor necrosis factor-α and matrix metalloproteinase-3 in periodontal tissue of diabetic rats

**DOI:** 10.3892/mmr.2015.3591

**Published:** 2015-04-02

**Authors:** YINGTAO WU, WANCHUN WANG, LIAN LIU

**Affiliations:** 1 Department of Periodontology and Oral Mucosa Diseases, Qingdao Stomatological Hospital, Qingdao, Shandong 266001, P.R. China; 2 Department of Acupuncture and Moxibustion, Qingdao Hiser Medical Group, Qingdao, Shandong 266001, P.R. China

**Keywords:** β-anhydroicaritin, diabetes, periodontal tissue, tumor necrosis factor-α, matrix metalloproteinase-3

## Abstract

The present study aimed to investigate the effect of β-anhydroicaritin on the expression levels of tumor necrosis factor (TNF)-α and matrix metalloproteinase (MMP)-3, and the pathological changes in the periodontal tissue of diabetic rats. Male Wistar rats (n=40; three months old) were randomly divided into four groups: Normal control group, diabetes group, diabetes + β-anhydroicaritin group and diabetes + urate group, (n=10 in each group). Following an overnight fast, diabetes was induced by intraperitoneal injection of streptozocin. The rats were maintained for 12 weeks and the blood sugar, urine sugar and body weight were assessed in week 12. Histological changes of the periodontal tissues were observed by hematoxylin and eosin staining, and the expression levels of TNF-α and MMP-3 were observed by immunohistochemistry. Following 12 weeks, the TNF-α grey value in the diabetes group was significantly lower compared with that in the control group (P<0.05), while no significant difference was observed between TNF-α levels in the diabetes + β-anhydroicaritin group, diabetes + urate group and the control group (P>0.05). However, TNF-α levels in the diabetes + β-anhdroicaritin group and diabetes + urate group were significantly higher compared with those in the diabetes group (P<0.05), and those in the diabetes + β-anhydroicaritin group were lower compared with those in the diabetes + urate group (P<0.05). The MMP-3 grey value in the diabetes group was significantly lower compared with that in the control group (P<0.05), while no significant difference was observed between MMP-3 levels in the diabetes + β-anhydroicaritin group, diabetes + urate group and the control group (P>0.05). However, MMP-3 levels the diabetes + β-anhydroicaritin group and diabetes + urate group were significantly higher compared with those in the diabetes group (P<0.05), and those in the diabetes + β-anhydroicaritin group were lower compared with those in the diabetes + urate group (P<0.01). β-anhydroicaritin normalized the expression levels of TNF-α and MMP-3 in the periodontal tissue of diabetic rats and led to the recovery of the changes in the morphological structure of the periodontal tissue.

## Introduction

Diabetes involves the state of chronic hyperglycemia caused by a range of genetic and environmental factors, and it is widely considered as one of the risk factors for periodontal disease (1). Previous studies have suggested that advanced glycosylation ends (AGEs) triggered by long-term hyperglycemia can stimulate phagocytes to release inflammatory cytokines, including tumor necrosis factor (TNF)-α, interleukin (IL)-1β and IL-6 (2), which may activate osteoclasts and matrix metalloproteinases (MMPs), and lead to the destruction of bone and periodontal tissue (3). TNF-α is one of the most important inflammatory factors and exerts a variety of biological effects. It can promote the degradation of connective tissue matrix and is the leading cytokine of local alveolar bone absorption, induced by inflammation (4). MMPs are tightly associated with periodontal tissue destruction and reconstruction, and are important in the development of periodontitis (5). MMP-3 is a member of the MMP family. It leads to the direct degradation of the extracellular matrix and also induces the expression levels of MMP-1, MMP-8 and MMP-9, and consequently causes more extensive damage in periodontal tissue (6). Therefore, the upregulation of the expression of MMP-3 is more significant compared with the upregulation of other MMPs. The alterations of TNF-α and MMP-3 in periodontal tissue may lead to changes and disorders in the periodontal tissue, promoting the development of periodontitis (7).

The present study established a rat model of diabetes and observed histological changes of the periodontal tissues under the microscope following tissue staining with hematoxylin and eosin. The expression levels of TNF-α and MMP-3 were observed by immunohistochemistry. The periodontitis induced by diabetes is closely associated with oxidative stress. Urate is an antioxidant, which is able to bind to ONOO- in normal conditions. It is considered to be a specific scavenger of ONOO- (8,9). Oxidative stress is an imbalance between the production of reactive oxygen species and antioxidant function, leading to tissue injury. The produced reactive oxygen species, such as superoxide and hydroxyl radicals, are able to damage numerous biological molecules, including DNA and lipid. It has been suggested that there is a causal association between insulin resistance, oxidative stress and periodontitis, and that hyperglycemia is a major cause of oxidative stress (10,11). The present study aimed to investigate the effect of β-anhydroicaritin on the changes in the expression levels of TNF-α and MMP-3, and pathological changes in the periodontal tissue of diabetic rats, providing a theoretical basis for the prevention of diabetic periodontitis.

## Materials and methods

### Reagents and animals

Wistar rats (n=40; three months old; body weight, 200–220 g) were purchased from Shanghai Slaccas experimental animal Co., Ltd. (Shanghai, China; certificate no. SCXK 2013-0012).

β-Anhydroicaritin was purchased from the National Institute for the Control of Pharmaceutical and Biological Products (Beijing, China). Urate was purchased from Shanghai Jimei Biotechnology Co. Ltd. (Shanghai, China). Streptozocin (STZ) was purchased from Hangzhou Baitong Biological Technology Co. Ltd. (Hangzhou, China). Rabbit anti-TNF-α polyclonal antibody (cat. no. ab6671) and rabbit anti-MMP-3 polyclonal antibody (cat. no. ab137659) were purchased from Abcam (Cambridge, MA, USA).

An Olympus BX51 fluorescence microscope was purchased from Olympus (Tokyo, Japan). The HPIAS-100 medical color picture analysis system was purchased from Wuhan Champion Image Engineering Company (Wuhan, China). A OneTouch ultra blood glucose meter was purchased from LifeScan (Milipitas, CA, USA).

### Animal groups and animal model

The present study was approved by the ethics committee of Qingdao University (Qingdao, China). The rats (n=40) were randomly divided into four groups: Normal control group, diabetes group, diabetes + β-anhydroicaritin group and diabetes + urate group (n=10 in each group). Following an overnight fast, diabetes was introduced by intraperitoneal injection of 30 mg/kg STZ diluted in citrate buffer (pH 4.4; Sigma-Aldrich, St. Louis, MO, USA).

At three days following STZ injection, the animals with blood glucose levels >15 mmol/l and urine glucose above +++ were considered as diabetic. Following the establishment of the diabetes models, the diabetes + β-anhydroicaritin group were administered 100 mg/kg/day β-anhydroicaritin and the diabetes + urate group were administered 100 mg/kg/day urate. All rats had free access to water and food, and were maintained under a 12:12 h light/dark cycle at 22±2°C and 65–69% relative humidity for 12 weeks. At week 12, the blood glucose, urine glucose and body weight were assessed (12).

### Specimen collection

Following being maintained for 12 weeks, the rats were euthanized by femoral artery bleeding following a 12 h-fast. The maxilla was extracted immediately and was fixed in 10% formaldehyde solution (Beyotime Biotechnology, Shanghai, China).

### Histological detection

The maxilla samples were placed in 0.01 M phosphate buffered saline (PBS; pH 7.4) for 12 h and the solution was renewed twice over this time-period. The samples were enucleated and fixed in formaldehyde, then processed and embedded in paraffin (Leica Microsystems, Wetzlar, Germany) using routine procedures (13). At the maxillary first molar tooth, along the long axis of the proximal and distal length, tooth periodontal slices (6 *μ*m) were cut.

The slices were dewaxed with xylene (Beyotime Biotechnology) and rehydrated with a descending graded series of alcohol. The slices were subsequently rinsed with tap water and hematoxylin-stained for 5 min. The slices were then rinsed, 1% diluted ammonia was added for 30 sec to retrieve the blue color, and the slices were again rinsed. The slices were stained with eosin for 5 min, rinsed, dehydrated with a graded series of alcohol, made transparent with xylene and finally mounted with neutral gum (Beyotime Biotechnology).

### Immunohistochemical analysis of TNF-α and MMP-3

Routine paraffin sectioning, dewaxing and hydration using 3% hydrogen peroxide (Beyotime Biotechnology) were performed to remove the endogenous peroxidase. Following this, ~50 *μ*l (1:50) rabbit polyclonal anti-TNF-α and rabbit polyclonal anti-MMP-3 (1:1,000) antibody were added, and the mixture was incubated at 4°C overnight. Following incubation, ~50 *μ*l biotinylated goat anti-rabbit immunoglobulin G secondary antibody (1:100; cat. no. A0277; Beyotime Biotechnology) working solution was added, the mixture was incubated at 37°C for 30 min, and colored using diaminobenzidine. The sample was stained with hematoxylin (Beyotime Biotechnology), separated using ethanol and hydrochloric acid (Beyotime Biotechnology), saturated with lithium carbonate (Sigma-Aldrich) until the color returned to blue, dehydrated with a graded alcohol series and xylene, and mounted with neutral gum.

The positive standard for immunohistochemistry was cytoplasmic and was stained yellow or brown. According to the color depth (HPIAS-100), staining intensity was scored as: Negative (−), not coloring; weakly positive (+), slight staining; positive (++), medium staining; strong positive (+++), deep staining.

For grey value determination, the color multimedia image analysis system was used to analyze the immunohistochemistry staining results of each specimen semi-quantitatively. At a magnification of ×200, a positive signal (grey value) was regarded in the first molar gingival and distal root in the 1/3 region of the periodontal membrane as parameter to analyze the results. The present study analyzed five sections and considered the average as the result. The grey background of each section was measured in order to eliminate the differences between sections.

### Statistical analysis

The data are expressed as the means ± standard deviation. SPSS 15.0 (SPSS, Inc, Chicago, IL, USA) was used for the statistical analysis. One-way analysis of variance was used to compare the data and the level of the test was a=0.05. Following determination of a statistically significant difference, further two-two comparison was performed. P<0.05 was considered to indicate a statistically significant difference.

## Results

### Confirmation of the diabetic model at three days following STZ injection

The average normal fasting blood glucose was 5.73 mmol/l, urine (−). The blood glucose level in the STZ injection rats was >15 mmol/l, urine (+++), which was significantly increased compared with that the normal group (P<0.01; Table I).

### Changes in the physiological features, body weight and blood glucose of the rats

The normal group demonstrated normal appearance and behavior, while the diabetes group appeared markedly thin and presented with a dull coat, unresponsiveness to stimuli, polydipsia, polyuria, polyphagia. The β-anhydroicaritin treatment group and the urate treatment group exhibited similar features to those in the diabetes group; however, these were milder compared with those in the diabetes group.

Comparison of fasting blood glucose and body weight in each group prior to and following the experiment revealed that at 12 weeks, the fasting blood glucose in the normal group exhibited no significant change (P>0.05) and the body weight increased significantly (P<0.05). The blood glucose in the diabetic group exhibited no significant change (P>0.05); however, the body weight significantly decreased (P<0.01). The blood glucose in the β-anhydroicaritin treatment group was significantly decreased (P<0.01) and the body weight also significantly decreased (P<0.01). In the urate treatment group, the blood glucose exhibited no significant change (P>0.05); however, the body weight was significantly decreased (P<0.01; Tables II and III).

Comparison of the levels of fasting blood glucose and weight in each group following the experiment revealed that the blood glucose levels in the diabetes group, the β-anhydroicaritin treatment group and the urate treatment group were significantly higher compared with those in the normal group (P<0.01), and the body weight was significantly lower compared with that in the normal group (P<0.01). The blood glucose levels in the β-anhydroicaritin treatment group were significantly lower compared with those in the diabetic group (P<0.01) and the urate treatment group (P<0.01). The blood glucose levels in the urate treatment group and the diabetic group were not significantly different from each other (P>0.05). Furthermore, the body weight of the β-anhydroicaritin treatment group, the urate treatment group and the diabetes group were not significantly different from each other (P>0.05; Table IV).

### HE staining

**Figure 1:**
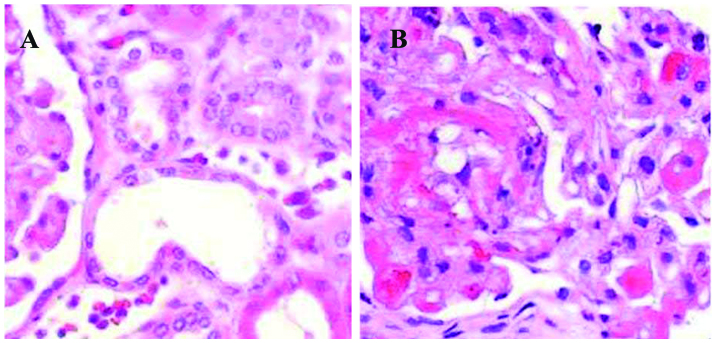
Hematoxylin and eosin staining of normal periodontal tissue. (A) Junctional epithelium in the control group. (B) Parodontium in the control group. Magnification, ×400.

In the control group, the junctional epithelium was attached to the cementumenamel junction and was firmly combined with the tooth tissue. No inflammatory infiltration was observed in the epithelium and the gingival and periodontal ligament fibers were arranged orderly and neatly. The alveolar bone edge was smooth and no osteoclast was observed. The alveolar ridge crest exhibited no absorption (Fig. 1).

**Figure 2:**
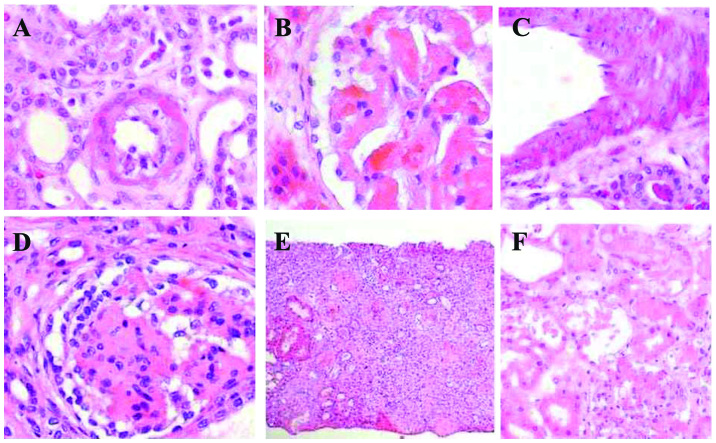
Hematoxylin and eosin staining of periodontal tissue in the diabetes group. (A) Junctional epithelium tissue. (B) Gingival epithelium tissue. (C) Inflammatory cells infiltrate gingival epithelium tissue. (D) Osteoclasts in alveolar bone. (E) Collagen of gingival tissue. (F) Collagen of periodontal tissue. Magnification, ×400.

The diabetes group exhibited junctional epithelium attached to the cementumenamel junction. The gingival epithelium and lamina propria exhibted moderate infiltration of inflammatory cells and the number of capillaries in the gingival and periodontal membrane increased. The capillaries demonstrated dilatation and congestion. Fibers were disordered and ruptured. The bone resorption lacunae along the alveolar bone increased and osteoclasts were observed. The alveolar ridge crest exhibited no absorption (Fig. 2).

**Figure 3:**
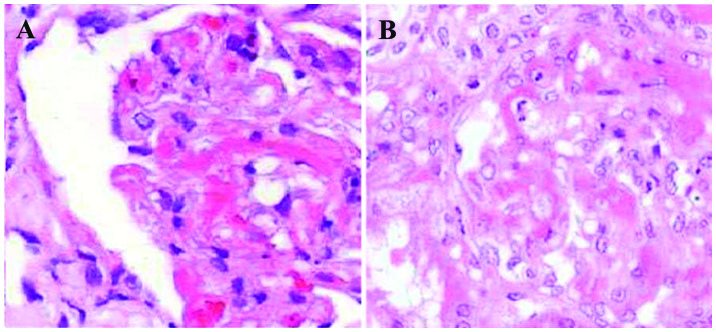
Hematoxylin and eosin staining of the periodontal tissue in the diabetes + β-anhydroicaritin group. (A) Junctional epithelium. (B) Parodontium. Magnification, ×400.

The diabetes + β-anhydroicaritin group exhibited junctional epithelium attached to the cementumenamel junction. The gingival epithelium and lamina propria demonstrated moderate infiltration of inflammatory cells. The capillaries exhibited no marked dilatation and congestion, and the fibers appeared regenerated and ordered. Bone resorption lacunae along the alveolar bone decreased. Osteoclasts were significantly reduced and the number of osteoblasts was increased as compared with those in the diabetes group. The alveolar ridge crest exhibited no absorption (Fig. 3).

**Figure 4:**
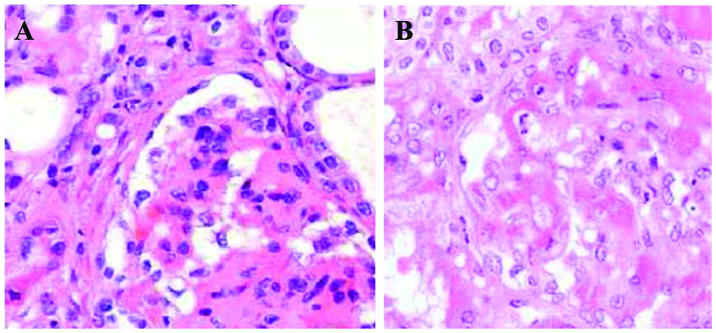
Hematoxylin and eosin staining of the periodontal tissue in the diabetes + urate group. (A) Junctional epithelium. (B) Parodontium. Magnification, ×400.

In the diabetes + urate group, the results were similar to those in the diabetes + β-anhydroicaritin group (Fig. 4).

### Expression levels of TNF-α and MMP-3 in the periodontal tissue

Immunohistochemical analysis showed that in the control group, the expression of TNF-α was weakly positive in the gingival epithelial layer, granular layer, spinous layer and basal layer cells, fibroblasts and the cytoplasm of endothelial cells. The expression of MMP-3 was predominantly weakly positive in fibroblasts and the cytoplasm of alveolar bone cells (mainly osteoblasts), and negatively expressed in the gingival epithelium (Table V).

**Figure 5:**
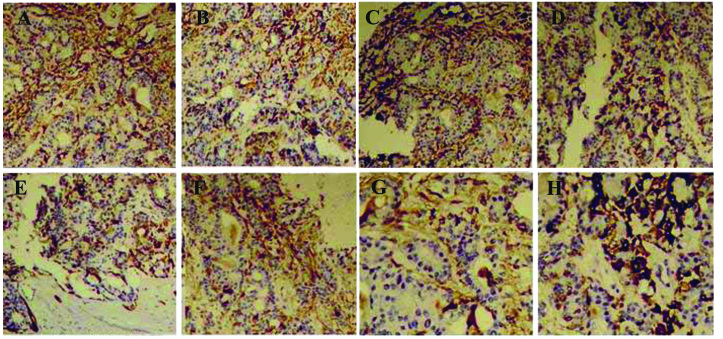
Histochemical analysis of TNF-α expression in the periodontal tissue in the diabetes group. There was positive expression of TNF-α in (A) the gingival epithelial layer, (B) the granular layer and stratum spinosum, (C) fibroblasts, (D) vascular endothelial cells, (E) osteoblasts, (F) osteoclasts, (G) the cytoplasm of bone marrow stromal cells and (H) the gingival epithelial basal layer. Magnification, ×400. TNF, tumor necrosis factor.

**Figure 6:**
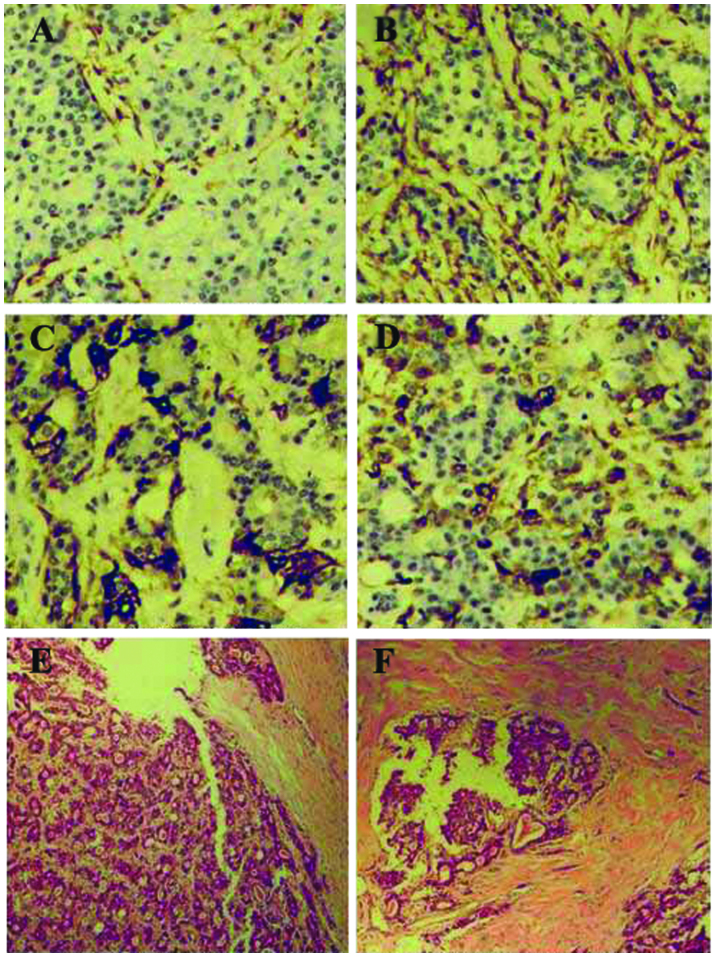
Expression of MMP-3 in the periodontal tissue in the diabetes group. MMP-3 was positively expressed in (A) the gingival epithelial layer, (B) the granular layer and stratum spinosum, (C) fibroblasts, (D) alveolar bone cells, (E) the cytoplasm of bone marrow stromal cells and (F) the gingival epithelial basal layer. Magnification, ×400. MMP, matrix metalloproteinase.

In the diabetes group, the expression of TNF-α was strongly positive in the gingival epithelial layer, granular layer and stratum spinosum cells, fibroblasts, vascular endothelial cells, osteoblasts, osteoclasts and the cytoplasm of bone marrow stromal cells, and was positively expressed in the gingival epithelial basal layer (Fig. 5). The expression of MMP-3 was strongly positive in the gingival epithelial layer, granular layer and stratum spinosum cells, fibroblasts, alveolar bone cells (mainly osteoclasts) and the cytoplasm of bone marrow stromal cells, and was negatively expressed in the gingival epithelial basal layer (Fig. 6).

**Figure 7:**
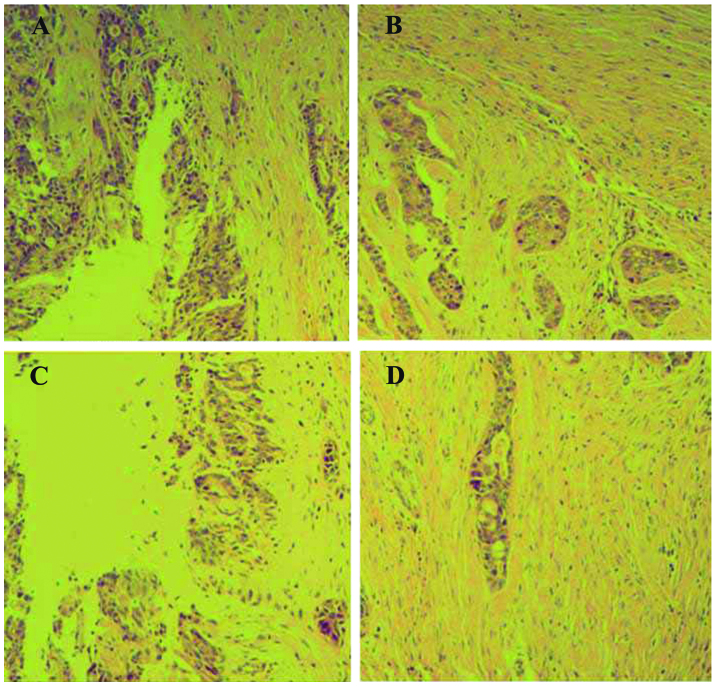
Expression levels of TNF-α and MMP-3 in the periodontal tissue in the diabetes + β-anhydroicaritin group. TNF-α was marginally positively expressed in (A) the gingival epithelial layer and (B) fibroblasts. MMP-3 was marginally positively expressed in (C) the gingival epithelial layer and (D) fibroblasts. Magnification, ×400. TNF, tumor necrosis factor; MMP, matrix metalloproteinase.

In the diabetes + β-anhydroicaritin group, the expression of TNF-α was weakly positive in the gingival epithelium and fibroblasts. The expression of MMP-3 was weakly positive in the gingival epithelium, fibroblasts and the cytoplasm (Fig. 7).

In the diabetes + urate group, the results were similar to those in the diabetes + β-anhydroicaritin group (results not shown).

The grey value of TNF-α was determined for all groups. The diabetic group exhibited a significantly lower grey value compared with that in the normal group (P<0.05). The β-anhydroicaritin treatment group exhibited no significant difference in TNF-α levels compared with those in the normal group (P>0.05); however, they were significantly higher compared with those in the diabetic group (P<0.05). The β-anhydroicaritin treatment group exhibited a lower grey value compared with that in the urate treatment group (P<0.05; Table V).

The grey value of MMP-3 in the diabetic group was significantly lower compared with that in the normal group (P<0.01). MMP-3 levels in the β-anhydroicaritin treatment group and urate treatment group exhibited no significant difference compared with those in the control group (P>0.05); however, they were significantly higher compared with those in the diabetic group (P<0.05). The β-anhydroicaritin treatment group exhibited a lower MMP-3 grey value compared with that in the urate treatment group (P<0.05; Table V).

## Discussion

STZ is a broad-spectrum antibiotic, which has anti-bacterial and anti-tumor properties, and causes side effects which lead to diabetes mellitus. STZ has highly selective toxic effects on islet cells in experimental animals, which leads to direct destruction of pancreatic β cells of the islet tissue or causes an immune response and decreases the insulin secretion of islets, which leads to diabetes (14,15). This model has advantages, including a simple preparation method, low dosage, low toxicity and specific islet cell damage, and it has become the preferred method of establishing a rat model of diabetes (16). The animals used to establish the model are usually inbred Wistar rats or Sprague Dawley rats. Daniel *et al* (17) reported that the success rate of model establishment in male rats was significantly higher compared with that in female rats. Monea *et al* (18) reported that the sensitivity of rats to STZ occurred in an age-dependent manner. The present study used male Wistar rats with an average body weight of 212.62 g. Following an overnight fast, diabetes was introduced by intraperitoneal injection of 30 mg/kg STZ diluted in citrate buffer (pH 4.4). At three days following the STZ injection, the blood glucose and urine glucose levels were assessed. The animals with blood glucose levels >15 mmol/l and urine glucose of +++ were considered as diabetic. At week 12, the blood glucose, urine glucose and body weight were assessed. The body weight of the diabetic rats was significantly reduced (P<0.01) and glucose levels were >15 mmol/l, which indicated that animals were diabetic from the establishment of the diabetic model until the end of the experiment.

Diabetes is a disease characterized by chronic high blood glucose and it is associated with genetic and environmental factors. It is due to a defect in insulin secretion and/or action, which causes disordered sugar, fat and protein metabolism (19,20). Patients with diabetes often suffer from periodontitis. Previous studies indicated that diabetes affected periodontitis incidence, severity and wound healing (21). Previous reports have demonstrated that rats exhibited alveolar bone resorption at one month of diabetes, which was aggravated with the extension of duration. The extension of bone resorption expanded, spreading to the alveolar bone to which the majority of periodontal fibers were attached, destroying the Haversian system and invading the bone marrow cavity (22,23). Demmer *et al* (24) found that bone resorption in the diabetic rat was present. The formation of new bone was slow and alveolar bone destruction exceeded bone repair.

The results of the present study suggested that junctional epithelium in the control group was attached to the cementumenamel junction and was firmly combined with the tooth tissue. No inflammatory infiltration was observed in the epithelium, and gingival and periodontal ligament fibers were arranged orderly and neatly. The alveolar bone edge was smooth and no osteoclasts were observed. The alveolar ridge crest exhibited no absorption. However, in the diabetes group, the junctional epithelium was attached to the cementumenamel junction, and the gingival epithelium and lamina propria exhibited moderate infiltration of inflammatory cells. The number of capillaries in the gingival and periodontal membrane increased, and the capillary demonstrated dilatation and congestion. The fibers were in disordered and ruptured, and bone resorption lacunae along the alveolar bone increased. Osteoclasts were observed and the alveolar ridge crest exhibited no absorption. These results suggested that diabetes alone was not able to cause periodontitis; however, it may destroy the normal structure of the periodontal tissue, which leads to a vulnerable state of the periodontal tissue. As a result, periodontitis is easily caused by a local stimulus.

TNF is one of the important inflammatory factors, which can be divided into two types: TNF-α and TNF-β. TNF-α is produced and secreted by several cells, including monocytes/macrophages, fibroblasts, vascular endothelial cells, osteoblasts, osteoclasts, T lymphocytes, smooth muscle cells and adipocytes (25,26). TNF-α can cause a variety of biological effects when acting on different target cells. When acting on fibroblasts, it can promote cell proliferation and the generation of prostaglandin 2; when acting on vascular endothelial cells, it can increase vascular permeability; and when acting on bone and cartilage tissue, it can cause bone absorption (27,28). These characteristics of TNF-α suggest that it is associated with the destruction of the connective tissue of the periodontal tissue and the alveolar bone absorption (29). MMP-3 (also termed stromelysin-1) is an important member of the MMP family, which is secreted by fibroblasts, endothelial cells and other connective tissue cells. It has an important role in tissue degradation, as its functions are to decompose protein polysaccharide and break down type I, V, IX, XI collagen, fibronectin and laminin (30,31). The predominant role of MMP-3 in the process of periodontal tissue destruction is that it directly degrades the extracellular matrix and causes more extensive destruction of the periodontal tissue through inducing the expression and activation of MMP-1, MMP-8 and MMP-9 (32). Therefore, increased expression of MMP-3 is more significant compared with the increased expression of other MMPs.

The expression levels of TNF-α and MMP-3 are low in normal periodontal tissue (33). The present study demonstrated that in the control group, the expression of TNF-α was weakly positive in the gingival epithelial layer, granular layer, spinous layer and basal layer cells, fibroblasts and cytoplasm of endothelial cells. The expression of MMP-3 was predominantly weakly positive in fibroblasts and the cytoplasm of alveolar bone cells (mainly osteoblasts), and negatively expressed in the gingival epithelium. The results indicated that normal periodontal tissue produced a certain amount of TNF-α and MMP-3 in order to ensure that the periodontal tissue remodeling was in a dynamic balance. Once the balance was disturbed, it led to structural changes in the periodontal tissue.

The high-permeability state of diabetes, AGEs and a variety of secreted cytokines induced by abnormal oxide are important in the pathophysiological process of the body and tissue damage caused by diabetes (34). Hyperglycemia leads to the non-enzymatic glycosylation of a group of proteins and lipids. They are combined with high-affinity receptors on the macrophage surface, triggering the secretion of TNF-α, IL-1β, IL-6 as well as other inflammatory mediators (35). At the same time, hyperglycemia leads to the upregulation of the expression levels of MMPs and nitric oxide. These factors may lead to bone absorption and the destruction of connective tissue (36).

The present study demonstrated that β-anhydroicaritin ameliorated the degradation of periodontal tissue and inhibited the synthesis and secretion of TNF-α and MMP-3 in diabetic rats. In conclusion, the results of the present study suggest that β-anhydroicaritin may be used in the treatment of periodontitis in patients with diabetes. However, the mechanisms by which β-anhydroicaritin ameliorates periodontal degradation, i.e. whether it directly inhibits or regulates the body’s metabolic function through the elimination of ONOO^−^ and further inhibits TNF-α and MMP-3 expression indirectly, remains to be elucidated. Further investigations are required to shed light upon these questions.

## Figures and Tables

**Table I:** Glucose levels in the blood and urine of rats.

Group	n	Blood glucose (mmol/l)	Urine glucose (mmol/l)
Control	10	5.73±0.61	−
Diabetes	10	19.74±1.82^a^	+++
Diabetes + β-anhydroicaritin	10	19.46±1.75^a^	+++
Diabetes + urate	10	19.83±1.78^a^	+++

a P<0.01, vs. control group.

**Table II:** Comparison of fasting glucose prior to and following the experiment.

Group	n	Glucose prior to experiment (mmol/l)	Glucose following experiment (mmol/l)
Control	10	5.73±0.61	5.62±0.58
Diabetes	10	19.74±1.82	19.53±1.67
Diabetes + β-anhydroicaritin	10	19.46±1.75	12.92±1.64^a^
Diabetes + urate	10	19.83±1.78	18.76±1.92

The values are expressed as the mean ± standard deviation.

a P<0.01, vs. prior to experiment.

**Table III:** Comparison of body weight prior to and following the experiment.

Group	n	Weight prior to experiment (g)	Weight following experiment (g)
Control	10	211.75±7.66	348.56±11.35^a^
Diabetes	10	209.16±8.34	162.91±9.54^b^
Diabetes + β-anhydroicaritin	10	210.62±7.92	154.68±8.38^b^
Diabetes + urate	10	210.68±7.84	158.83±9.49^b^

The values are expressed as the mean ± standard deviation.

a P<0.05 and

b P<0.01, vs. prior to experiment.

**Table IV:** Comparison of fasting glucose and body weight following the experiment.

Group	n	Blood glucose (mmol/l)	Body weight (g)
Control	10	5.62±0.58	348.56±11.35
Diabetes	10	19.53±1.67^a^	162.91±9.54^a^
Diabetes + β-anhydroicaritin	10	12.92±1.64^a^,^b^,^c^	154.68±8.38^a^
Diabetes + urate	10	18.76±1.92	158.83±9.49^a^

The values are expressed as the mean ± standard deviation.

a P<0.01, vs. control group;

b P<0.01, vs. diabetes group;

c P<0.01, vs. urate group.

**Table V:** Grey values of TNF-α and MMP-3 in the periodontal tissue.

Group	n	TNF-α	MMP-3
Control	10	97.67±24.65	82.66±17.61
Diabetes	10	73.24±21.41^a^	52.14±16.32^b^
Diabetes + β-anhydroicaritin	10	95.17±23.28^c^	63.25±16.64^c^
Diabetes + urate	10	108.61±26.92^d^,^e^	93.35±18.47^d^,^f^

The values are expressed as the mean ± standard deviation.

a P<0.05,

b P<0.01, vs. control group;

c P<0.05,

d P<0.01, vs. diabetes group;

e P<0.05,

f P<0.01, vs. diabetes group. TNF, tumor necrosis factor; MMP, matrix metalloproteinase.
